# Modulation of the leptin receptors expression in breast cancer cell lines exposed to leptin and tamoxifen

**DOI:** 10.1038/s41598-019-55674-x

**Published:** 2019-12-16

**Authors:** Rodolfo López Linares, Jonnathan Guadalupe Santillán Benítez, Mariana Ortiz Reynoso, Carlos González Romero, Antonio Sandoval-Cabrera

**Affiliations:** 1 Facultad de Química, Universidad Autónoma del Estado de México (UAEMex), Toluca, México; 2 Laboratorio de alta especialidad en Hemato-Oncología, Hospital para el niño, IMIEM, Toluca, México

**Keywords:** Biochemistry, Biological techniques, Cancer, Molecular biology, Molecular medicine, Oncology

## Abstract

One of the factors that has increased the incidence and worse prognosis of breast cancer is obesity. In this condition, high amounts of leptin are secreted, which have proliferative, mitogenic, antiapoptotic, and proinflammatory activity that may be antagonistic to treatment with tamoxifen, considered the first choice. The modulation evaluation of leptin receptor expression in the presence of leptin and tamoxifen stimuli was performed in breast cancer cell lines MCF 7, MDA MB 231 and HCC 1937 as a model of initial approach for the study of breast cancer subtypes and their behavior to the action response of adipokines and their possible relationship with the mechanism of resistance to chemotherapeutics such as tamoxifen in ER positive cell lines and triple negative marker. It was determined that leptin increases the proliferation of the three breast cancer cell lines and tamoxifen is able to exert an antiproliferative effect on them, however, it was identified that the ability of tamoxifen to decrease the proliferation of cancer cells is diminished in the presence of leptin, in addition to changes in the modulation of the expression of its receptor. It was determined that tamoxifen induces a greater modulation of the expression of ObRb in cell lines, which may be related to the decrease of its antiproliferative activity, while leptin generates a proliferative effect in the three cell lines and could participate in the tamoxifen treatment resistance mechanism.

## Introduction

Breast cancer is the main type of neoplasm that affects women worldwide; it is a pathology that can be triggered due to multiple factors that lead to an abnormal and uncontrolled proliferation of the cells that make up the mammary gland, which is constituted by three different cell types: myoepithelial, ductal epithelial cells and alveolar epithelial cells^1^.

In turn, breast cancer can be classified into different subtypes depending on the expression pattern of some receptors, such as estrogen (ER), progesterone (PR) and human epidermal growth factor type 2 (Her-2), giving rise to luminal breast cancer A (ER+, PR+), luminal B (ER+, PR+), Her-2 positive and triple negative marker. This classification is very useful to identify the type of chemotherapeutic treatment that should be administered, as well as the degree of risk and prognosis of the condition^2^.

Obesity is defined as the abnormal or excessive accumulation of fatty tissue in the body, is a health problem that has received worldwide attention in recent years as its incidence has increased and it is associated as a risk factor for the development of chronic degenerative diseases that include cardiovascular diseases, diabetes mellitus type 2, hypertension and some types of cancer, including breast cancer^3,4^.

There are different mechanisms by which obesity has been linked to cancer, among which are the promotion of inflammation and angiogenesis through the release of some molecules by adipocytes such as fibroblast growth factor (FGF2), angiopoietin-2, angiostatin, endostatin, thrombospondin (TSP-1), resistin, and elevated levels of adipokines such as leptin, among others^5^.

Leptin is a polypeptide hormone that is produced in adipose tissue, whose plasma levels are increased in patients who are obese, generating a condition known as hyperleptinemia^6^. Under normal physiological conditions, leptin maintains energy homeostasis by influencing the central anorectic pathway, where it acts to reduce food intake and increase metabolism^7^.

On the other hand, it also participates in the regulation of pathological processes such as breast cancer by presenting mitogenic, antiapoptotic and proinflammatory activity, and it is only capable of inducing the proliferation of cancer cells and not of normal breast cells, which is indicative that leptin participates directly in the development of this type of cancer^8,9^.

To exert its biological function, leptin has a specific receptor (ObR), which has six different isoforms, which vary in the amount of intracytoplasmic residues they contain. Among them, ObRb, also known as the long isoform of the leptin receptor, is considered to be the one with the greatest capacity to activate signaling pathways^10^.

Within the signaling pathways activated by leptin are: JAK2-STAT3, mitogen-activated protein kinase (MAPK) and phosphatidylinositol 3-kinase/protein kinase B (PI3K/AKT), which generates the activation of some genes such as: C-MYC, CYCLIN D1, P21 WAF1, C-JUN, JUNB, ERG-1 and BCL-2, which are strongly involved in cell growth and proliferation^4,11^.

The relationship between obesity, leptin and breast cancer has taken a further slope, that of chemotherapy resistance mediated by adipokines, proteins released by adipose tissue and can be involved in the action of chemotherapeutic drugs, since the higher risk of recurrence and mortality in patients with obesity and breast cancer could be related to a lower efficacy of cancer treatments, this is probably due to the plasma variations of adipokine levels, since women with breast cancer and obesity are less sensitive to chemotherapy and have higher mortality rates^7^.

In relation to the above, it has been determined that leptin can influence the development of resistance to treatment with tamoxifen, an antiestrogenic drug commonly administered to breast cancer patients with the presence of ER. It was observed that combination therapy involving the blockade of the leptin receptor and treatment with tamoxifen could inhibit proliferation and promote apoptosis in breast cancer cells resistant to tamoxifen, suggesting that the suppression of leptin could be a new means to circumvent resistance to antiestrogenic treatment in breast cancer, because the signaling pathways activated by leptin could directly participate in the cellular response to the chemotherapeutic treatment^12^.

Therefore, the objective of this work is to evaluate the modulation of gene expression of leptin receptors in three different breast cancer cell lines as an initial approach model for the study of the behavior of subtypes of this disease before its exposure to leptin and tamoxifen.

## Materials and Methods

### Cell culture

The cell lines MCF-7, MDA-MB 231 and HCC1937 were obtained from the American Type Culture Collection (ATCC). The cells were cultured at 37 °C in a 5% CO2 atmosphere, with RPMI 1640 culture medium (RPMI Medium 1640, Gibco Thermo Fisher Scientific, USA) supplemented with 10% FBS (Fetal Bovine Serum, certified, US origin, Gibco Thermo Fisher Scientific, USA) and 1% penicillin-streptomycin. Additionally, for the MCF-7 and MDA-MB 231 cell lines, the culture medium was enriched with a 2 mM pyruvate concentration and for the HCC 1937 line, a 2 mM concentration of glutamine was used. The culture medium was changed every third day.

### IC50 determination of tamoxifen

Proliferation of 5,000 cells of the cell lines MCF-7, MDA-MB 231 and HCC1937 (n = 3) was carried out in 96-well plates for 48 hours in RPMI-1640 medium (10% SFB). Subsequently they were stimulated for 48 hours with the following concentrations of tamoxifen: 0.002 μM, 0.02 μM, 0.2 μM, 2 μM, 20 μM and 200 μM, changing the stimulus every 24 hours.

At the end of the 48 hours of stimulation, the cells were fixed with 4% formaldehyde for 15 minutes. The fixed cells were washed with PBS and stained with crystal violet (0.02%) for 15 minutes, to remove the residual dye, 3 washes of 250 μl of water were carried out and finally 250 μl of 10% acetic acid was added to each well. The plate was read at an excitation wavelength of 490 nm and an emission length of 630 nm in a microplate reader (Stat Fax 2100 Microplate Reader, Awareness Technology Inc.).

### Proliferation assays

Proliferation of 5,000 MCF-7 cells, MDA-MB 231 and HCC1937 (n = 3) was carried out in 96-well plates for 48 hours. The cells were then washed with PBS and fresh culture medium (supplemented to 5% of SFB) was added with leptin stimulation (Recom Hu Leptin Active, Sino Biological, Thermo Fisher Scientific, USA) in concentrations of 0 ng/mL, 10 ng/mL, 100 ng/mL and 200 ng/mL for 72 hours, changing the stimulus every 24 hours. Additionally, another test was carried out using these concentrations of leptin plus a corresponding standard with the IC50 of tamoxifen (Tamoxifen, Sigma-Aldrich) obtained experimentally for each of the cell lines (MDA MB 231: 2,230 μM, MCF7: 10,045 μM, HCC 1937: 4.579 μM) and another only with the tamoxifen standard. The decrease in SFB was performed to decrease growth factors that could interfere with the action of leptin and tamoxifen^13^.

Every 24 hours after the stimulation the cells were fixed with 4% formaldehyde for 15 minutes. At the end of the 72 hours of stimulation, the fixed cells were stained with crystal violet (0.02%) for 15 minutes, to remove the residual dye, 3 washes of 250 μl of water were carried out and finally 250 μl of 10% acetic acid was added to each well. The plate was read at an excitation wavelength of 490 nm and an emission length of 630 nm in a microplate reader (Stat Fax 2100 Microplate Reader, Awareness Technology Inc.).

### Gene expression analysis

The regulation of gene expression of leptin receptors was evaluated by adding leptin and tamoxifen in cell lines, using the following methodology proposed by Jardé *et al*.^14^, with some modifications.

20,000 cells of MCF-7, MDA-MB 231 and HCC1937 (n = 3) were plated in 24-well plates for 48 hours. The stimulation was carried out with leptin (10 ng/mL, 100 ng/mL and 200 ng/mL) and tamoxifen (HCC 1937: 4.579 μM, MDA MB 231: 2230 μM and MCF 7: 10.045 μM) in culture medium RPMI-1640 (5% SFB) for 72 hours with stimulus change every 24 hours. At the end of the stimulation, the culture medium was removed and 750 μL of TRIzol Reagent was added to carry out the RNA extraction according to the protocol of use (Ambion).

Once the RNA was obtained, complementary DNA (cDNA) was obtained from 2 μg of mRNA by means of the reverse transcriptase reaction according to the protocol of use (High Capacity RNA-to-cDNA kit, Applied Biosystems) obtaining 20 μL of cDNA. The same was used for the amplification of ObRb, ObRt, ER, aromatase and 18S control genes under initial amplification conditions at 94 °C for 5 minutes, 35 cycles (94 °C for 30 seconds, 57 °C for 30 seconds, 72 °C for 60 seconds) and a final extension at 72 °C for 10 minutes, using the HotStarTaq DNA Polymerase, (QIAGEN, Germany) and the following pairs of primers: ObRb (Fw: 5′-GATAGAGGCCCAGGCATTTTTTA-3′, Rv: 5′-ACACCACTCTCTCTCTTTTTGATTGA-3′), ObRt, (Fw: 5′-CATTTTATCCCC ATTGAGAAG TA-3′, Rv: 5′-CTGAAAATTAAGTCCTTGTGCCCA-3′), ER (Fw: 5′-GTGTACAACTACCCCGAGGGC-3′, Rv: 5′-AAACCCCCCAGGCCGTTGGAG-3′) and aromatase (Fw: 5′-CAAGGTTATTTTGAT GCATGG-3′, Rv: 5′-AATCCTTGACAGACTTCTCAT-3′), gene 18S (Fw: 5′-GTC TGTGATGCCCTTAGA TG-3′, Rv: 5′-AGCTTATGACCCGCACTTAC-3′).

Likewise, the cDNA obtained was used to perform the determination of gene expression of leptin receptors by real-time PCR (TaqMan Universal Mix II, with UNG) using TaqMan probes (Gene Expression Assays, Applied Biosystems) for ObR (Hs00174497_m1) and as control gene on 18S (Hs99999901_s1) following the instructions of the LightCycler 2.0 thermocycler (Roche Diagnostics) for each sample in duplicate. The program used to perform the RT-PCR was 50 °C for 2 minutes, 95° for 10 minutes and 40 cycles at 95 °C for 15 seconds and 60 °C for 1 minute. For the determination of the degree of genetic expression of the leptin receptors, the relative quantification method of Pfaffl was used, based on the comparison of the CT of a constitutive gene (18S) against the problem gene taking the value of its differences against the control line for each sample^15^.

### ELISA

It was performed using the supernatants of the stimulated cell cultures and under the protocol of use of the ELISA kit for human leptin receptor (Human Leptin receptor ELISA Kit. DEIA8050 Creative Diagnostics). The detection range of the test ranges from 156 to 10,000 pg/mL. The plate was read with the microplate reader (Stat Fax 2100 Microplate Reader, Awareness Technology Inc.) at a wavelength of 450 nm.

### Statistic analysis

The statistical analysis was performed using ANOVA for the growth curves of each cell line. The value of P < 0.05 will be considered as statistically significant.

For tests of gene expression, the normality test was used using the Kolmogorov-Smirnov test. Subsequently, Student’s t test for parametric variables and the Mann-Whitney U test for nonparametric variables with a p value > 0.05 for each one were performed.

### Ethical approval

This article does not contain any studies with human participants or animals performed by any of the authors.

## Results

### Leptin, estrogen and aromatase receptors mRNA expression in breast cancer cell lines

**Figure 1:**
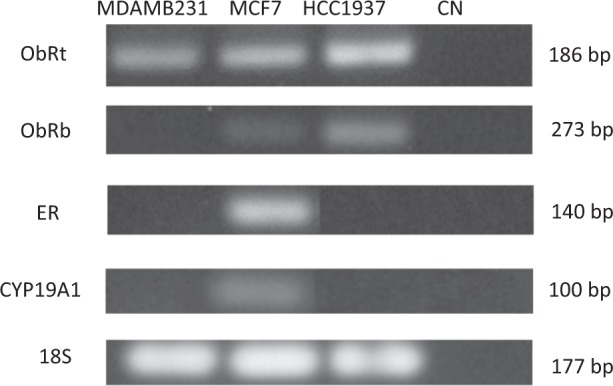
Electrophoresis in agarose gel 1.5% stained with BrEt of the RT-PCR products. The bands obtained for each gene of interest (ObRt, ObRb, ER, CYP19A1) and a negative control (CN) in each cell line (MDA MB 231, MCF7 and HCC1937) are shown, as well as the size of the amplified sample.

The determination of the constitutive expression of mRNA was carried out by RT-PCR of the long isoform of the leptin receptor (ObRb), homologous region for any of the six different isoforms of the leptin receptor (ObRt), estrogen receptor (ER) and aromatase (CYP19A1) in the three breast cancer cell lines prior to stimulation with leptin and tamoxifen, as shown in Fig. 1.

In the MCF7 cell line, the presence of mRNA of the four genes of interest was found, in the MDA MB 231 cell line only mRNA of the general homologous form of the leptin receptor was found and in the HCC 1937 cell line the expression of the leptin receptor was detected in its two variants.

### Determination of the IC50 of tamoxifen for each cell line

**Figure 2:**
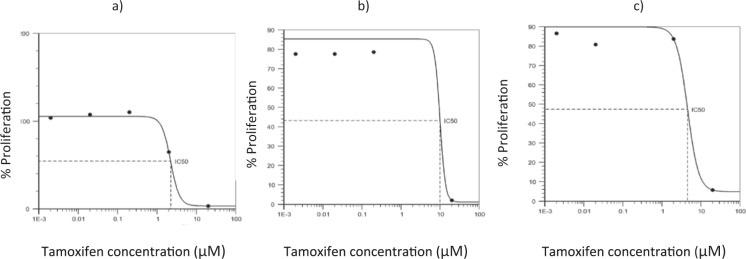
Graphs of the determination of tamoxifen IC50 in breast cancer cell lines. The dose response curves obtained for each cell line with the tamoxifen stimulus are shown. In graph (**a**) the IC50 is observed of tamoxifen for the MDA MB 231 cell line corresponding to 2230 μM, in graph (**b**) the IC50 is observed for the cell line MCF 7 corresponding to 10.045 μM and in the graph (**c**) the one corresponding to the HCC 1937 cell line whose concentration is 4,579 μM is observed.

From the proliferation assay a dose response curve was obtained for each cell line (Fig. 2) from which the IC50 of tamoxifen was determined, resulting in 2,230 μM for the MDA line MB 231, 10,045 μM for the MCF7 line and 4,579 μM for the HCC 1937 line.

### Leptin and tamoxifen effect on the proliferation of breast cancer cell lines MDA MB 231, MCF 7 and HCC 1937

**Figure 3:**
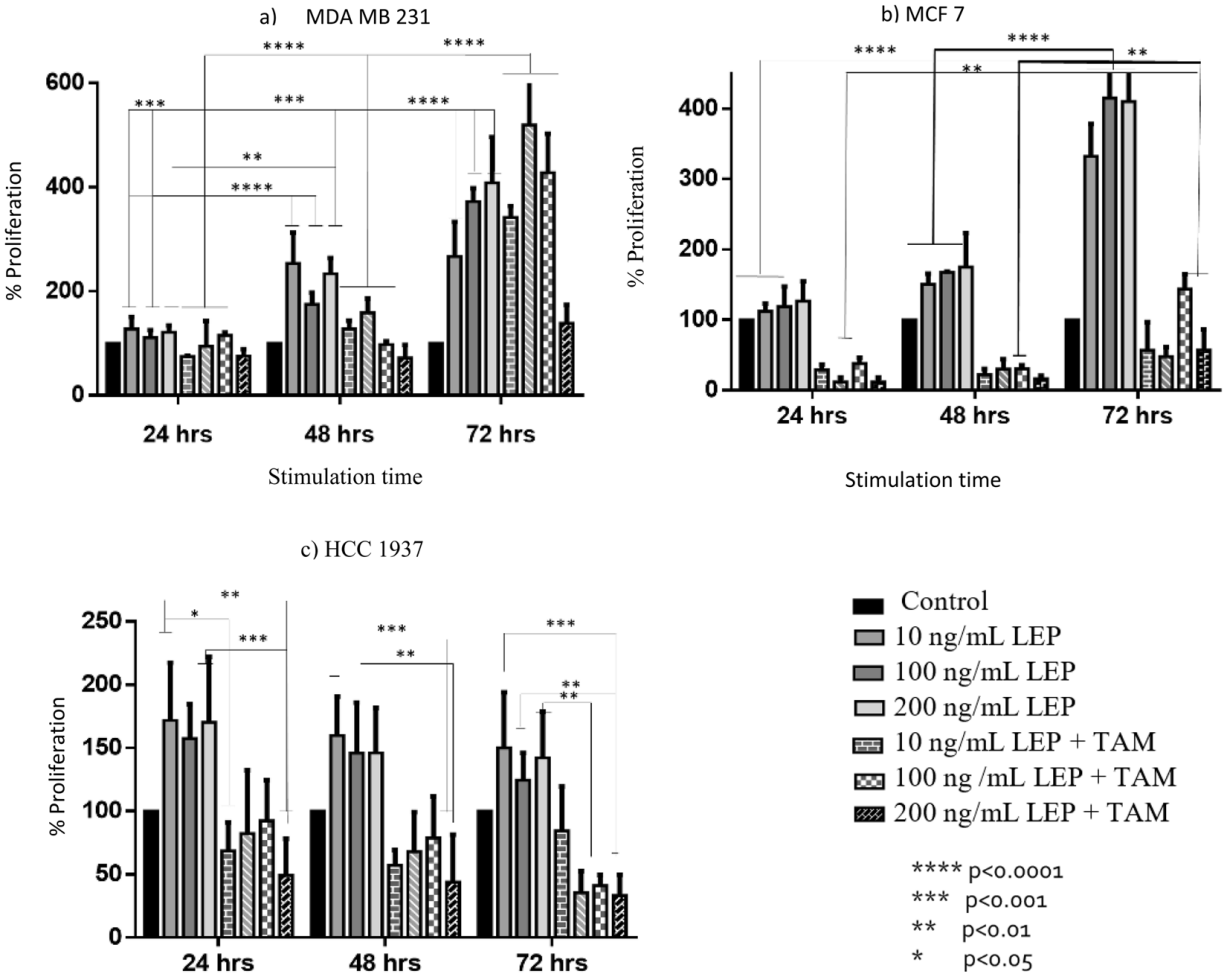
Effect of leptin and tamoxifen on the proliferation of breast cancer cell lines. Figure (**a**) shows the effect presented in the MDA MB 231 cell line, in figure (**b**) the effect in the cell line MCF 7 and in figure (**c**) the effect produced in the HCC cell line 1937 is shown.

In general, in the cultures of the three different breast cancer cell lines, a positive proliferative effect was observed by stimulation with leptin, the opposite effect was observed when stimulating with tamoxifen (Fig. 3).

The cells were cultured for 48 hours and subsequently stimulated with leptin and tamoxifen, observing the following results: in the MDA MB 231 cell line stimulation of leptin in any concentrations causes a proliferative effect from 24 hours of stimulation (Fig. 3, sub-figure a), reaching an increase in the cell population of 27.18% for 10 ng/mL, 10.75% for 100 ng/mL and 21.12% for 200 ng/mL, this increase in cell proliferation was increased by the time of exposure to the leptin stimulus, reaching the greatest effect at 72 hours, finding an increase of 166.6% for 10 ng/mL, 272.22% for 100 ng/mL and 308.33% for 200 ng/mL.

In the cell line MCF7 (Fig. 2, sub-figure b) an increase in cell proliferation was observed when stimulated with leptin in the three concentrations during the 72 hours, reaching an increase in proliferation of up to 232.22% for 10 ng/mL, 315% for 100 ng/mL and 310% for 200 ng/mL after 72 hours of stimulation compared to the control. In this cell line the action of tamoxifen is more evident, which reduced the cell population in the three stimulation times, however, it can be observed that when tamoxifen is administered in combination with leptin, cell proliferation is greater than with the group only exposed to tamoxifen, similarly it is observed that the greater effect of tamoxifen, occurs during the first 48 hours of stimulation.

In the cell line HCC 1937 (Fig. 3, sub-figure c) an increase in cell proliferation was observed when stimulated with leptin, reaching a maximum effect at 24 hours of stimulation with an increase of 71.74% for 10 ng/mL, 57.3% for 100 ng/mL and 70.3% for 200 ng/mL compared to the control, subsequently a decrease in proliferation is observed. In this cell line tamoxifen decreases proliferation, however, the same effect is seen as the two previous cell lines, where when leptin is combined with tamoxifen, cell proliferation is greater than where it is only stimulated with tamoxifen. In this cell line, there are no statistically significant differences between the different stimulation times.

### Expression of the leptin receptor in breast cancer cell lines MDA MB 231, MCF 7 and HCC 1937 exposed to leptin and tamoxifen

**Figure 4:**
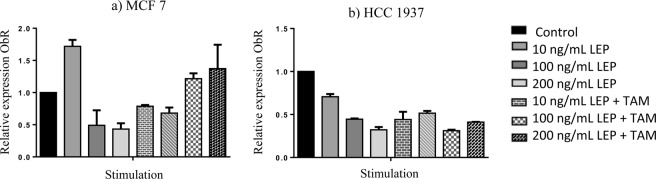
Relative expression of ObR. Graphs showing the relative expression of ObR, in the cell lines MCF7 (**a**) and HCC1937 (**b**) obtained by real-time PCR.

The relative expression of leptin receptor mRNA (ObR) was determined in the different cell lines as shown in Fig. 4, obtaining the following results.

In the MCF-7 cell line, the expression of the ObR had an increase of 71.86% (10 ng/mL) in the cells treated with leptin with respect to the control, however, at concentrations of 100 and 200 ng/mL of leptin, a decrease in ObR expression is observed, for the stimulation of tamoxifen a 37.2% increase in the expression of the gene was observed, with respect to the control, while for the combination stimuli of leptin with tamoxifen a decrease in the expression of the gene was observed.

In the HCC 1937 cell line, a decrease in all cases of ObR expression was identified.

In the MDA MB 231 cell line, no amplification was obtained with the Taqman probe for the leptin receptor.

### Determination of ObRt protein by the ELISA technique

**Figure 5:**
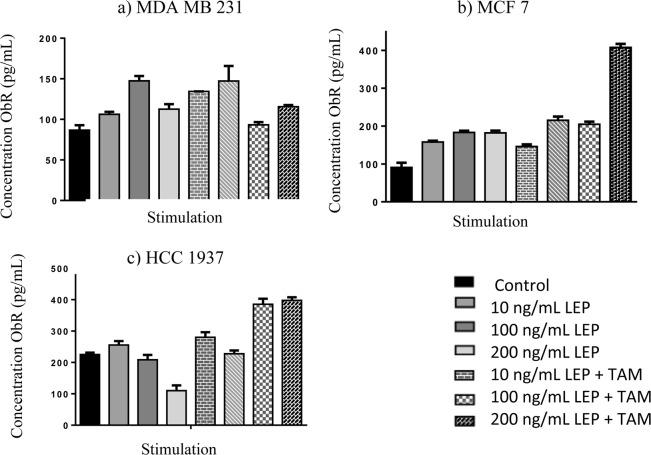
Concentration of the ObR protein in breast cancer cell lines. Graphs showing the concentrations of the ObRt protein obtained by the ELISA test, in figure (**a**) the determination for the MDA line MB 231 is observed, in figure (**b**) for the MCF line 7 and in the figure (**c**) for the HCC 1937 line.

The determination and quantification of the leptin receptor protein in the supernatant of each of the cultures stimulated by the ELISA technique was carried out, obtaining the results shown in Fig. 5.

In the MDA MB 231 cell line, a higher concentration of the ObR protein was found in all the groups of stimulated cells compared to the control, reaching the highest concentration in stimuli that include 100 ng/mL of leptin and 100 ng/mL of leptin plus tamoxifen, with a concentration of 147.3 pg/mL.

In the MCF 7 cell line, greater expression of the ObR protein was found in all cases where stimulus was added, however, it is of great interest that the stimulus that showed a higher concentration of the protein was in which only tamoxifen was added, obtaining a concentration of 407.7 pg/mL, while in the trials that include leptin, something similar to the MDA MB 231 line is found, since the stimuli that generated the highest expression of the protein were those of 100 ng/mL of leptin and 100 ng/mL of leptin plus tamoxifen, with concentrations of 183.1 pg/mL and 215.3 pg/mL respectively.

In the HCC 1937 cell line, greater expression of the ObR protein was found in the stimuli corresponding to 200 ng/mL of leptin plus tamoxifen and only tamoxifen, where the concentration of 385.7 pg/mL and 397.5 pg/mL respectively was reached. A different behavior was identified in the stimulus corresponding to 200 ng/mL of leptin since a decrease in the expression of the ObR protein was observed, finding a concentration of 110.1 pg/mL which is lower than the concentration identified in the control which was 225.3 pg/mL.

## Discussion

The determination of mRNA expression by RT-PCR was determined for the long isoform of the leptin receptor (ObRb), homologous region for any of the six different isoforms of the leptin receptor (ObRt), estrogen receptor (ER), aromatase (CYP19A1) and the 18s gene in the three breast cancer cell lines, as part of the characterization prior to stimulation with leptin and tamoxifen, it was determined that in the MCF7 cell line the mRNA of the five genes of interest is expressed, which is consistent with the description of this cell line, since as an identification feature it expresses the estrogen receptor and therefore the presence of CYP19A1 is found, an enzyme that participates in the estrogen biosynthesis, likewise the presence of leptin receptor mRNA was found, both in the homologous form for any of the six different isoforms of the receptor, as for the long isoform (ObRb), which is of interest since it is considered as the isoform that has greater activity and capacity to activate signaling pathways such as JAK2/STAT3 due to the intracytoplasmic residues it presents^2^.

In the MCF7 cell line, the maximum increase in the proliferation of cancer cells was identified when stimulated with leptin for 72 hours, after a growth prior to stimulation of 48 hours, reaching an increase in proliferation of 232.22% for 10 ng/mL, 315% for 100 ng/mL and 310% for 200 ng/mL, which indicates that leptin generates an increase in the proliferation of this cell line and that after 100 ng/mL a significant increase is no longer found, the increase in cell proliferation corresponds to the reported characteristics of the activation of leptin signaling pathways, which contribute to the progression of cancer through multiple mechanisms, since it is known that leptin activates several signaling cascades, such as the JAK/STAT, MAPK and PI3 kinase/AKT pathways, most of which are involved in the differentiation and proliferation of cancer cells. In addition to that it has been shown that treatment with leptin suppresses apoptosis and accelerates the progression of the cell cycle through the induction of genes related to it^16^.

In this cell line a greater anti-proliferative effect of tamoxifen is observed in comparison with the other cell lines (MDA MB 231 and HCC 1937), reaching a 76.12% decrease in cell proliferation, this is because tamoxifen is an antiestrogenic drug that acts by binding with the estrogen receptor competing with estrogen and nullifying the biological effects of these molecules, the tamoxifen-estrogen receptor complex interacts with nuclear DNA and blocks the action of estrogen by inhibiting the production of growth factors, which negatively affects cell proliferation^17^.

There are other types of cell lines that belong to the type of triple negative breast cancer, which is an aggressive form that is characterized by the absence of some membrane receptors such as ER, PR and Her-2, which leads to limited treatment options. Therefore, in order to understand the molecular basis of this type of cancer, the analysis of the *in vitro* behavior of triple negative cell lines is crucial, in this study the MDA MB 231 and HCC 1937 lines were used.

In the MDA MB 231 cell line, neither the presence of estrogen receptor mRNA nor CYP19A1 was found, this is consistent with the characteristic of the cell line belonging to the triple negative cancer type, for which it lacks the expression of the estrogen, progesterone and Her-2 receptor. Regarding the presence of leptin receptor mRNA, it was found only for the general homologous form of the receptor, so it can not be established which of the six different isoforms of the receptor is the one that is being expressed in this cell line.

In the HCC 1937 cell line, which possesses a mutation in the BRCA1 gene, the presence of mRNA expression of the leptin receptor was found in the two isoforms analyzed (long and homologous isoform), as in the MDA MB 231 cell line, no expression of the estrogen receptor or CYP19A1 was found, as it was also a negative triple-negative breast cancer cell line.

In the two lines of cancer triple negative marker an increase in cell proliferation was identified when stimulated with leptin, which suggests that as in the cell line MCF 7, leptin participates activating signaling pathways that give this cell type greater proliferative capacity, one of these pathways is related to Wnt/β-catenin, which is key in the development of several types of cancer, since Wnt/β-catenin signal transduction fulfills fundamental functions in the regulation of the proliferation and differentiation of cancer cells. Activation of the Wnt/β-catenin pathway is enriched in different types of breast cancer. In a recent study Liang *et al*.; they identified results similar to those obtained in this study when they found an increase in cell proliferation in lines MCF 7 and MDA MB 231 when stimulated with leptin and an increase in β-catenin levels was also identified^18^.

**Figure 6:**
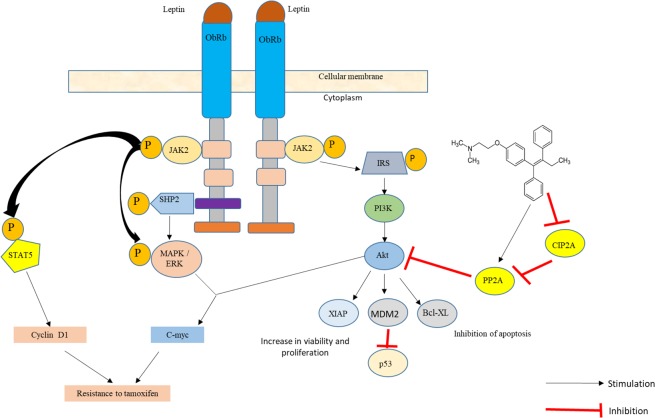
Proposal of tamoxifen resistance mechanism in breast cancer cell lines triple negative marker. Tamoxifen may exert antiproliferative action on the triple negative marker cell lines by stimulating the expression of PP2A, a tumor suppressor protein and inhibiting the activity of CIP2A, which favors the proliferation of cancer cells. There are different signaling pathways by which leptin could participate in the development of resistance to treatment with tamoxifen, including JAK2/STAT5, MAPK/ERK, PI3K/AKT and the activation of genes such as C-myc, XIAP, MDM2, Bcl -XL, etc^2,19,21–31^.

In both negative triple marker cell lines it was identified that tamoxifen generates a cytostatic effect on cell proliferation, which is interesting since tamoxifen is an antiestrogenic drug and its molecular target is the estrogen receptor, which is absent in these cell lines, however recent studies have identified that tamoxifen shows a response rate of 10% to 15% in tumors without ER expression, these findings suggest that tamoxifen has certain anticancer properties independent of ER. One of these mechanisms is the one that involves the decrease of the regulation of the cancerous inhibitor of protein phosphatase 2A (CYP2A) and p-Akt, which correlates with tamoxifen-induced apoptosis in ER-negative breast cancer cells, the possible mechanism that contemplates the interaction between leptin and tamoxifen in these cell lines is shown in Fig. 6 ^19^.

Regarding the relative expression of the ObR in the cell lines determined by qPCR, it was found that in the cell line MCF 7 there is an increase in the expression of the receptor in a 71.86% compared to the control for the concentration of 10 ng/mL of leptin, for the concentrations of 100 and 200 ng/mL the expression is surely diminished by a blockade of the expression due to the saturation of the receptor by the action of leptin, however it is important to note that when the cell line is stimulated with tamoxifen there is also an increase in the expression of the receptor, this is possibly due to the fact that the cancer cells induce a greater expression of the leptin receptor to maintain its viability and proliferation, since it has been determined that breast cancer cells use the signaling pathways of leptin as a fundamental part of maintaining their proliferation^20^.

In the HCC 1937 cell line, a decrease in ObR expression was found in all stimulation cases compared to the control, this is due to the fact that upon finding a higher expression of the ObR protein in this cell line, mRNA expression is diminished, whereas for the MDA MB 231 cell line the ObR amplification was not identified by qPCR, this is because the probe used does not amplify the desired sequence in this cell line, since it is focused on the detection of ObRb, which by RT-PCR was determined in MCF 7 and HCC1937 cell lines, but was not detected in MDA MB 231.

The ObR protein levels obtained by the ELISA technique show that for MCF 7 and MDA MB 231 cell lines, higher concentration was obtained compared to the control in all the stimuli, whereas in the HCC 1937 cell line an increase in the concentration of the protein was obtained when stimulated with leptin (10 ng/mL) and with leptin plus tamoxifen in its different concentrations, in the case of the stimulation of leptin at 100 and 200 ng/mL, a lower concentration of the protein was obtained compared to the control, this makes it possible to define that the expression of the ObR protein is expressed under normal physiological conditions and not in a marked increase in the concentration of leptin. It is important to note that in this study it was determined that in the three cell lines a higher concentration of ObR protein expression is observed when stimulated with tamoxifen, this probably corresponds to the ability of ER positive (MCF 7) and triple negative marker cells (HCC 1937 and MDA MB 231) to increase the expression of ObR as a survival mechanism against the damage caused by the chemotherapeutic agent^20,21^.

The presence of the ObR in the three cell lines corresponds to that reported in the literature where it is mentioned that the ObR is necessary for the maintenance and renewal of cancer cells^21^, however, it is of great interest the evidence found that when the tamoxifen stimulus is placed in combination with leptin in the three cell lines, greater cell proliferation is observed than when only tamoxifen is added, so it can be defined that probably the plasma variations of leptin levels in women with breast cancer and obesity participate in generating lower sensitivity to chemotherapy and this leads to higher mortality rates.

## Conclusions

In this study it was determined that leptin favors the proliferation of cell lines MCF 7, HCC 1937 and MDA MB 231, noticing a time-dose dependent effect; it was also determined that tamoxifen is able to generate its antiproliferative effect in the three cell lines, being the most noticeable effect in the cell line MCF7, which is ER positive.

It is observed that the combination of the leptin-tamoxifen stimulus generates a decrease in the antiproliferative capacity of tamoxifen, which leads to propose that leptin participates in the mechanism of resistance to tamoxifen treatment, commonly observed in patients with breast cancer and obesity.

Similarly, in this study it can be observed that the administration of tamoxifen in the three cell lines favors a higher expression of the ObR at the protein level, however, at the level of mRNA, the same can not be confirmed, since a greater expression is observed in the MCF7 cell line, while in the HCC1937 cell line, a decrease in the expression of the receptor is observed and in the MDA MB 231 cell line could not be determined because in this cell line the amplified one was not obtained by the Taqman probe.

## Supplementary information

Supplementary Information

Supplementary Information 3

Supplementary Information 4
